# ASO Author Reflections: Genetic and Immunohistochemical Studies Investigating the Histogenesis of Neuroendocrine and Carcinomatous Components of Combined Neuroendocrine Carcinoma

**DOI:** 10.1245/s10434-019-07880-0

**Published:** 2019-11-11

**Authors:** Misaki Iijima, Takehiko Yokobori, Akira Mogi, Ken Shirabe, Hiroyuki Kuwano

**Affiliations:** 1 Department of General Surgical Science, Graduate School of Medicine, Gunma University, 3-39-22 Showamachi, Maebashi, 371-8511 Japan; 2 Department of Innovative Cancer Immunotherapy, Graduate School of Medicine, Gunma University, 3-39-22 Showamachi, Maebashi, 371-8511 Japan; 3 Division of Integrated Oncology Research, Gunma University Initiative for Advanced Research (GIAR), 3-39-22 Showamachi, Maebashi, 371-8511 Japan

## Past

Lung combined neuroendocrine carcinomas (NECs) comprise NEC components such as small cell lung carcinoma (SCLC) or large cell NEC, and non-NEC components such as adenocarcinoma (ADC) or squamous cell carcinoma. Some researchers have reported that combined NECs have common epidermal growth factor receptor (EGFR) mutations in both NEC and non-NEC components,^1^,^2^ suggesting that these two components might originate from cells of the same origin. Interestingly, it has been reported that EGFR mutations are often observed in non-NECs, but are very rare in sporadic NECs, which almost always have p53 mutations.^3^ Moreover, a case report showed that lung ADC with EGFR mutation transforms to SCLC as an NEC component in the process of acquiring EGFR-tyrosine kinase inhibitor (TKI) resistance.^4^ Therefore, it was suggested that genetic and immunohistochemical analysis of EGFR and p53 for each component of combined NECs would provide important information on whether such components originate from the same tumor cells or incidentally arise as collision cancers.

## Present

We analyzed tumor specimens from eight patients with combined NECs who underwent surgical resection.^5^ The mutation status of EGFR and/or p53 was consistent between the NEC and non-NEC components in seven of eight cases (87.5%). Immunohistochemical analysis showed that synaptophysin expression as NEC markers was detected in all NEC components, but not in non-NEC components. We found that, in combined NECs in the lung, the NEC often harbors EGFR mutations that correspond to those in the non-NEC component, and replacement transformation occurs in the borderline area between non-NECs and NECs. Moreover, the activated EGFR signal found in non-NEC components was shown to be downregulated in NEC components.

## Future

Our study reports on the mechanism behind the carcinogenesis of lung combined NECs, which is caused partially by the transformation from epithelial carcinomas of non-NECs to NECs. This carcinogenic mechanism might be different from that in sporadic NECs without non-NEC components. Past studies have reported that several cancer-related genes, including p53 and Notch, play important roles in the carcinogenesis of NECs.^6^ Therefore, further study is needed to clarify the importance of these factors as a trigger for the replacement transformation in combined NECs. Moreover, NEC components of combined NECs with EGFR mutations did not share the non-NEC characteristics, such as EGFR activation. In future, we propose that the combination of chemotherapy against NEC and EGFR-TKIs against non-NECs may be a suitable therapeutic strategy in patients with EGFR-mutated combined NECs.
